# User experience of a hepatitis c population management dashboard in the Department of Veterans Affairs

**DOI:** 10.1371/journal.pone.0285044

**Published:** 2023-05-02

**Authors:** Vera Yakovchenko, David A. Jacob, Shari S. Rogal, Timothy R. Morgan, Karine Rozenberg-Ben-Dror

**Affiliations:** 1 Center for Healthcare Organization and Implementation Research, VA Bedford Healthcare System, Bedford, MA, United States of America; 2 Center for Health Equity Research and Promotion, VA Pittsburgh Healthcare System, Pittsburgh, PA, United States of America; 3 Veteran Affairs Heart of Texas Health Care Network, Temple, TX, United States of America; 4 Departments of Medicine and Surgery, University of Pittsburgh, Pittsburgh, PA, United States of America; 5 Gastroenterology Section, VA Long Beach Healthcare System, Long Beach, CA, United States of America; 6 Department of Medicine, Division of Gastroenterology, University of California, Irvine, CA, United States of America; 7 Veteran Affairs Great Lakes Health Care System, Westchester, IL, United States of America; Florida Atlantic University Charles E Schmidt College of Medicine, UNITED STATES

## Abstract

**Background:**

The Veterans Health Administration (VA) is the largest integrated healthcare organization in the US and cares for the largest cohort of individuals with hepatitis C (HCV). A national HCV population management dashboard enabled rapid identification and treatment uptake with direct acting antiviral agents across VA hospitals. We describe the HCV dashboard (HCVDB) and evaluate its use and user experience.

**Methods:**

A user-centered design approach created the HCVDB to include reports based on the HCV care continuum: 1) 1945–1965 birth cohort high-risk screening, 2) linkage to care and treatment of chronic HCV, 3) treatment monitoring, 4) post-treatment to confirm cure (i.e., sustained virologic response), and 5) special populations of unstably housed Veterans. We evaluated frequency of usage and user experience with the System Usability Score (SUS) and Unified Theory of Acceptance and Use of Technology 2 (UTAUT2) instruments.

**Results:**

Between November 2016 and July 2021, 1302 unique users accessed the HCVDB a total of 163,836 times. The linkage report was used most frequently (71%), followed by screening (13%), sustained virologic response (11%), on-treatment (4%), and special populations (<1%). Based on user feedback (n = 105), the mean SUS score was 73±16, indicating a good user experience. Overall acceptability was high with the following UTAUT2 rated from highest to least: Price Value, Performance Expectancy, Social Influence, and Facilitating Conditions.

**Conclusions:**

The HCVDB had rapid and widespread uptake, met provider needs, and scored highly on user experience measures. Collaboration between clinicians, clinical informatics, and population health experts was essential for dashboard design and sustained use. Population health management tools have the potential for large-scale impacts on care timeliness and efficiency.

## Introduction

Hepatitis C virus (HCV) infection is a leading cause of cirrhosis, liver cancer, and liver transplants in the US [1,2]. As the largest integrated healthcare system in the US, the Veterans Health Administration (VA) has had a longstanding public health goal of eliminating HCV [3]. Since the approval of HCV direct-acting antivirals (DAAs) in late 2013, the VA has cured over 115,000 Veterans, making VA the first healthcare system to approach HCV elimination in the US [4]. This system-wide successful implementation effort was led by VA’s HIV, Hepatitis, and Related Conditions Programs in the Office of Specialty Care Services and implemented by the national Hepatic Innovation Team Collaborative (Collaborative) [5]. The Collaborative employed quality improvement and system redesign strategies and leveraged VA’s fully integrated electronic medical record (EMR) and data warehouse to create a population health management (PHM) dashboard [6].

Dashboards are central to PHM approaches taken by the VA [3]. An important aspect of healthcare dashboards’ success is the user experience. User-centered design is an emerging field focused on engaging end users in designing products and tools to meet their needs. Fundamental principles of user-centered design include early stakeholder engagement and iterative testing and improvement which benefit early adoption, implementation, and sustained use [7].

The HCV dashboard (HCVDB) is a visual display of real-time data using business intelligence that empowers clinicians to make informed clinical decisions and rapidly scale up linkage and access to care. We have previously shown that the HCVDB was a widely used implementation strategy for increasing HCV treatment across VA; however, little has been published about use of the dashboard or user experience [8,9]. This study aims to describe the design, development, implementation, and evaluation of VA’s national HCVDB.

## Materials and methods

### Setting and participants

The VA is a large integrated healthcare system with over 1200 medical centers and community-based outpatient clinics serving nine million enrolled Veterans annually. As of 2014, VA had screened about 35% of 1945–1965 birth-cohort Veterans for HCV and cured 10% of Veterans with chronic HCV [4].

Participants were primary users of the HCVDB including physicians, advanced practice providers, and clinical pharmacists in liver and infectious disease clinics. Per VA Handbook 1058.05/Program Guide 1200.21 [10], this quality improvement project was conducted as a non-research operations activity for the HIV, Hepatitis, and Related Conditions Programs in the Office of Specialty Care Services as a part of the program evaluation for the Collaborative.

### Dashboard design

**Fig 1:**
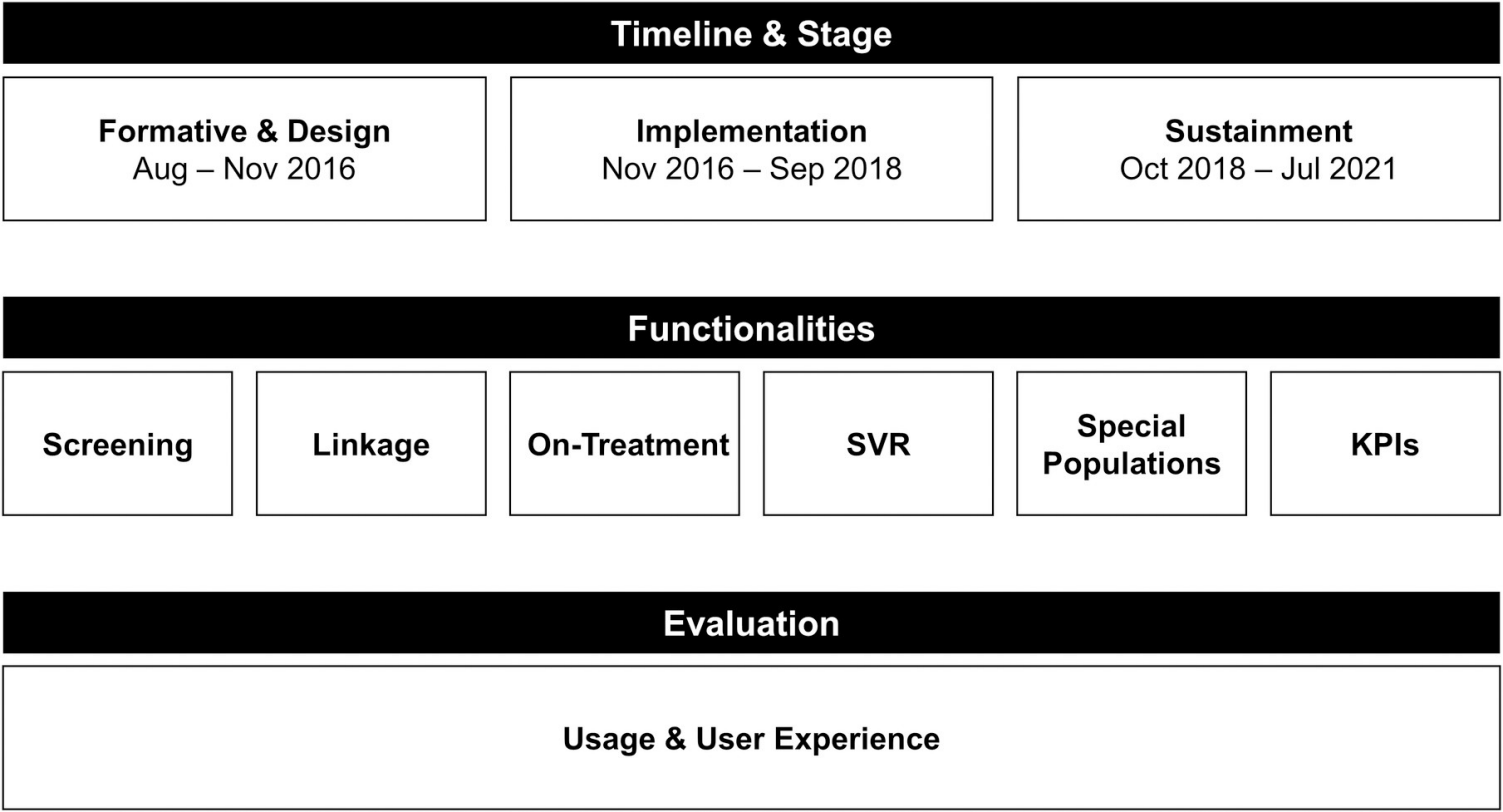
Dashboard development & evaluation.

A formative assessment preceded dashboard design (Fig 1). In August 2016, the VA held its second annual Collaborative Face-to-Face Meeting with liver care providers and system redesign staff from across 130 VA medical centers (facilities). A recurrent theme emerged during townhall-style discussions identifying a significant barrier due to lack of information technology tools to inform about the HCV continuum of care. Several facilities utilized local innovations to rapidly expand HCV treatment using PHM tools that were unavailable at facilities with lower uptake of HCV treatment. Consequently, and to address this data availability barrier, several early innovators with locally or regionally developed tools collaborated to develop a single national HCVDB to standardize and reduce duplication efforts and overcome disparities in data and reporting availability. The group became widely known as the HCV dashboard development team.

The dashboard development team was led by two Collaborative clinical pharmacy providers (KR, DAJ) who previously developed dashboards in their networks, and several other clinical pharmacy specialists with extensive clinical informatics and population health training. The group utilized user-centered design principles to review existing dashboards and selected features important for visual clarity and clinical decision support (Table 1) [11]. Two development team members (KR, DAJ) tested dashboard functionalities real-time at their facilities’ clinics to find Veterans with HCV and optimized linkage to care before engaging other facilities in dashboard user acceptance testing.

**Table 1:** Dashboard design.

**Elements of Clinical Decision Support:** • Rapid report rendering • Visual Display—a single visual view of all information on one screen • Real time • Accurate data (limited only to useful information) • Easy to incorporate into workflow or clinic process • Enable user to find their patients within a facility • Easy to learn across clinical users–Nurse Practitioners, Clinical Pharmacists, Physicians • Enable tracking of clinical decisions–free text • Provide accounting—Summary report for self-monitoring performance • Development team maintain clinical knowledge base—keep report current • Development team responds rapidly to provider question / Assistance • Development team includes a mechanism for feedback
**Unique features:** • Include data not easily found in the medical record • (Other VA facilities, include health factors/data elements) • Color flags to reduce provider fatigue and facilitate rapid scanning of important information • High FIB-4, low platelets, diagnosis of hepatocellular carcinoma • Facilitate user actions: • Sort patients into intervention groups • Waiting for labs • waiting for patient response • Unable to reach • Sort patients by next appointment • Display only patients needing interventions (needing review or late for labs)

This initial phase of development took approximately three months with a significant amount of time devoted to identifying and validating labs across all VA facilities (HCV antibody, genotype, viral load (VL), alanine aminotransferase (ALT), aspartate transferase (AST), platelets), and the first version of the national HCVDB was launched in November 2016 to allow for rapid treatment uptake. A similar process occurred real-time for inclusion of other functionalities in the report such as monitoring laboratory treatment response, as well as post-treatment achievement of HCV cure. Importantly, the developers of the report continued to use the report in clinic, monitor the data for quality, and implement changes to the report as new medications became available with shorter treatment durations and monitoring parameters. The Collaborative’s regional teams and the developers responded to issues from new users in the field, incorporating feedback from users throughout the process.

### Dashboard data

The HCVDB provides real-time SQL-based reports derived from the VA Corporate Data Warehouse using ICD-9/10 diagnoses from clinical encounters, laboratory tests, and pharmacy treatment data. Four technical reports within the dashboard target distinct national metrics across the HCV care continuum: screening, linkage, treatment, and post treatment. The fifth report stratifies Veterans by housing instability. The sixth report generates a real-time “score card” or summary report for key performance indicators (KPIs). Reports can be generated at multiple levels (national, regional, facility or provider-specific, etc.).

#### HCV unscreened birth cohort report

Based on earlier CDC guidelines for screening patients in the high-risk birth cohort (those born between 1945–1965), this report identifies patients needing HCV screening by provider, facility, region, and provides national-level testing rates for this population. This report was revised in 2020 to include the expanded CDC testing guidelines for adults 18–79 years old [12].

#### Linkage to HCV treatment report

**Fig 2:**
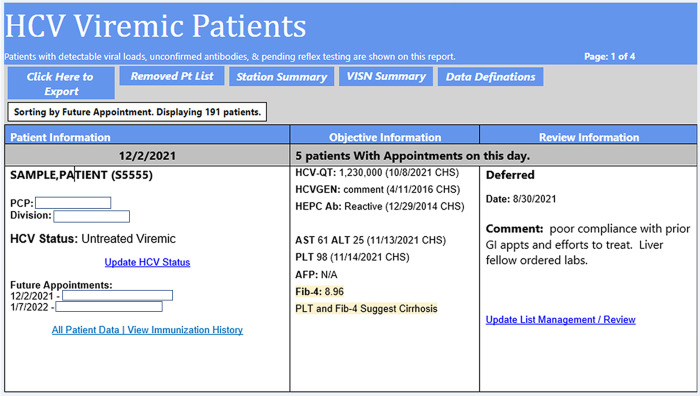
HCVDB screenshot.

This report identifies patients in care at the facility with chronic HCV viremia (with or without prior HCV treatment relapse). The report can be sorted by the next scheduled clinic appointment (primary care (PC), gastroenterology (GI), or infectious diseases (ID)) to enhance communication with clinic providers for patient engage for treatment on or near their next appointment time. Fig 2 shows a screenshot of a patient’s status with a next appointment date.

A sub-report in the linkage to HCV treatment report called *Patients with Recently Diagnosed Hep C* was developed for newly diagnosed patients which enables rapid identification and linkage to care. The sub-report is a line list of patients with new HCV viremia in the last 30 days, 90 days, or fiscal year. The report can filter out patients initiated on DAA for rapid review.

Linkage to treatment reports display the next two pending appointments with PC, GI or ID and can be sorted by appointment date or facility. Data in the report is compartmentalized into three sections: 1) Patient demographics and future appointments, 2) Objective HCV evidence (recent VL, genotype, ALT/AST/Platelet, fibrosis scores (FIB-4 and APRI) and a flag for advanced liver disease (ALD) and hepatocellular carcinoma), and 3) Provider comments for assessment of treatment candidacy.

Reviews could be entered directly in the report or generated from the EMR utilizing facility-based data objects pertaining to HCV. Patient treatment candidacy was determined by providers with expertise in HCV management, documented in the medical record, and tracked in the dashboard through a list management feature (a set of 15 standardized classifications in a drop-down menu such as treatment candidate, pending work-up, not a treatment candidate, deferred, follow up, or no treatment). In addition to selecting from the 15 options, a free text field allowed providers to include pertinent details as a quick way to distinguish between Veterans who were pending treatment initiation from those with ongoing treatment barriers (e.g., documented ongoing nonadherence, limited life expectancy, patient refusal). The comment field could be used to further distinguish between temporary or permanent barriers to treatment to enable repeat reviews at appropriate time intervals for patients who may later become treatment candidates (e.g., patient out of state for the winter, incarcerated, or pending surgery).

#### On-treatment report

The on-treatment report displays patients receiving DAA treatment at their facility from initiation through post-treatment determination of cure (i.e., sustained virologic response (SVR)) 12 to 24 weeks after completing therapy (SVR12, SVR24). It also contains color-coded flags to easily identify patients overdue for SVR testing or in whom viremia is detected during or after treatment completion (i.e., Red = Detectable RNA, Yellow = No Result, and Green = RNA Undetected). Patients can be sorted alphabetically or by treatment initiation date. SVR is resulted if an undetected VL occurs 10 or more weeks after completing treatment.

#### SVR report

A report was created to find patients that received DAA treatment without documentation of SVR or relapse. This report was requested to separate tasks related to patient care and help with concerted efforts to find patients who were lost to follow-up or needed to be re-engaged for finalized testing. The report is a line list of patient names, prior DAA treatment and the last known HCV VL. The SVR report also includes sorting and list management functionalities for tracking.

#### Special populations report

This report features Veterans with housing instability without HCV testing or with HCV viremia for treatment assessment. Veterans with a U.S. Department of Housing and Urban Development-VA Supportive Housing Program encounters in the last 90 days are displayed in the report to improve treatment outreach through social workers when the patient is stably housed and ready to embark on successful treatment.

#### Other iterations

As DAA treatment durations changed, report iterations were implemented to capture 8 weeks instead of 12 weeks of therapy. Some patients initiated their treatment later than the prescribed date which could lead to inconsistency in determining the true SVR date. Providers were able to edit the start date to ensure the report was capturing and flagging information correctly.

### Dashboard implementation

#### Training

User training for the HCVDB was a multipronged approach leveraging the Collaborative infrastructure to reach providers. First, an online data dictionary and guidebook/toolkit were created. Virtual group and one-on-one trainings were provided monthly and on demand to regions, facilities, and providers.

Providers reviewed their own patients during orientation to the HCVDB to maximize use of time and make orientation relevant to the providers receiving training. The Collaborative implemented a train-the-trainer approach by identifying superusers in each region to lead in training new users in other facilities. The HCVDB developers were available to troubleshoot with providers, which contributed to real-time resolution of data-related issues and complemented provider workflow and efficiency.

### Evaluation methods

#### Usage

We obtained usage analytics, including HCVDB users and counts of report executions and list management actions, between November 2016 and July 2021. The period November 2016 to September 2018 was considered “implementation” and October 2018 to July 2021 “sustainment.” Report execution units were counted based on opening report pages linked to a user’s VA identification. Reports could be downloaded (exported as an Excel file) and used without further interacting with the HCVDB, therefore we did not measure the amount of time the reports were used. List management involved provider sorting of patients into an intervention group and tracking a comment in their records.

#### User experience survey

An online survey was emailed to the Collaborative distribution list to identify providers who used the HCVDB or might be able to forward the invitation to others. Data were collected from September to December 2018. The survey asked about length of time using the HCVDB, primary purpose of use, level of importance of different reports and features, usability, and acceptability. A free-text comment box was included at the end of the survey. Respondent demographic information were collected including specialty, degree, and years of involvement with the Collaborative.

*Usability* was captured on the survey with the System Usability Scale (SUS), a 10-item valid and reliable measure of perceived usability on a five-point Likert scale from “strongly disagree” to “strongly agree” [13]. SUS scores are on a 0–100 scale with scores below 50 indicating a not acceptable level of usability, 50–70 marginal acceptability, and above 70 an acceptable level of usability. The SUS is graded on a curve, therefore a 68 is equivalent to about 50%, a score of 74 is about 70%, and 80 is the top 90%.

*Acceptability* was evaluated with the Unified Theory of Acceptance and Use of Technology 2 (UTAUT2) instrument. The UTAUT2 was abbreviated to include seven items measuring constructs of Performance Expectancy (n = 3), Social Influence (n = 1), Facilitating Conditions (n = 2), and Price Value (n = 1) [14]. UTAUT2 items were on a five-point Likert scale from disagree to strongly agree. A mean score for each domain and a summary score were calculated. Higher scores indicate more positive perceptions. The internal consistency reliabilities were .75 or greater and the average variance scored suggested discriminant validity [15].

### Analysis

Descriptive statistics were used to characterize participants, HCVDB usage, usability, and acceptability. Kruskal-Wallis tests analyzed differences in HCVDB use by staff and facility characteristics. Correlations examined the relationship between usage and user experience. Analyses were conducted with RStudio version 1.0.153. Free text comments were analyzed using thematic analysis and codes developed inductively from the data.

## Results

### Usage

**Fig 3:**
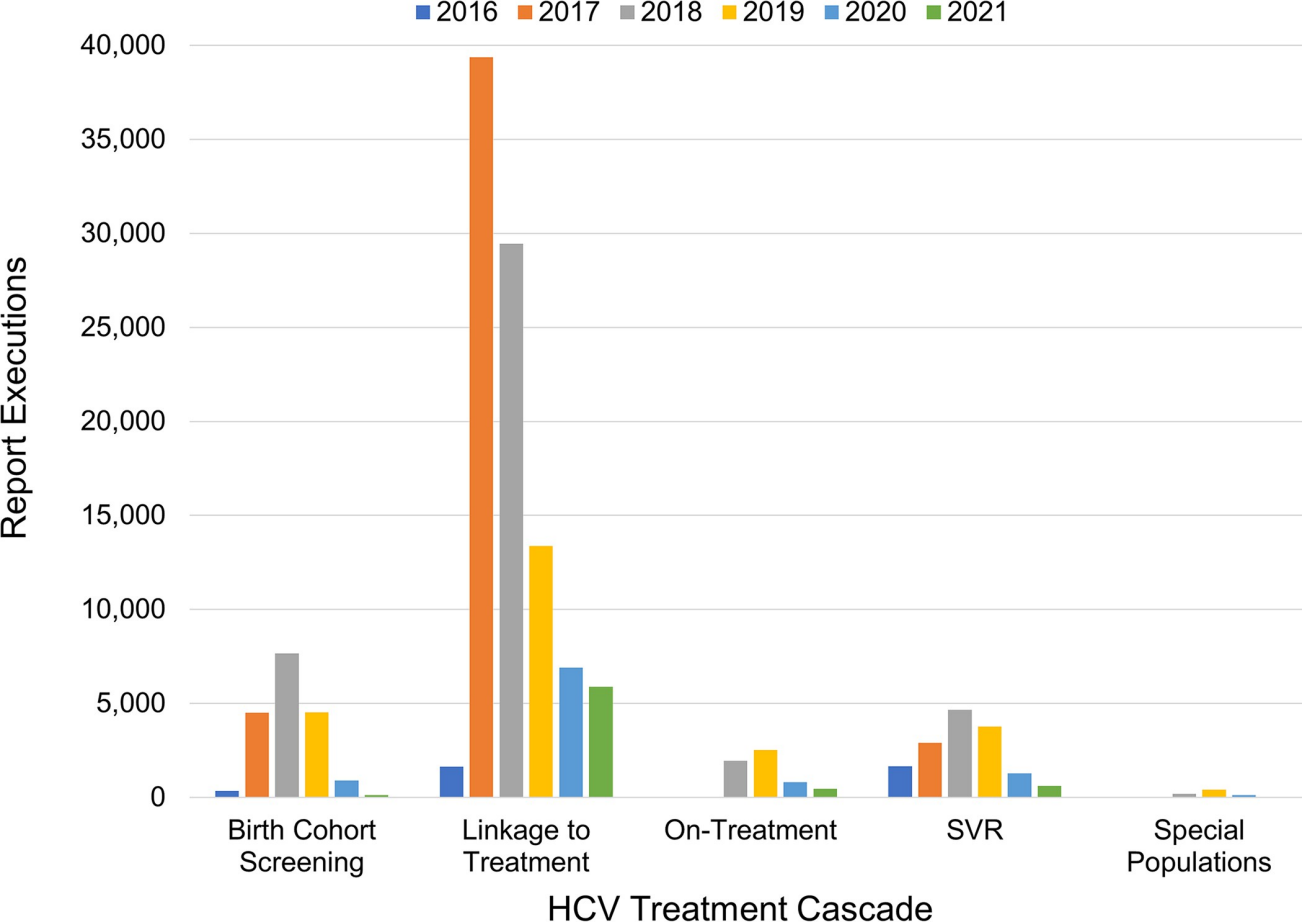
HCVDB use by report type.

Between November 2016 and July 2021 there were 163,836 report executions of HCVDB reports by 1302 users. The number of users was strongly positively associated with report executions (r = .84, p < .001). The majority of report executions occurred during the implementation period (2016–2018) compared to sustainment (2019–2021) (72% vs 28%). Of the five technical reports, the linkage to treatment report was used most frequently (71%) followed by screening (13%), SVR (11%), on-treatment (4%), and special populations (<1%). The KPI score card had over 27,000 executions. Fig 3 displays report use by year. The Linkage report in 2017 and 2018 accounted for 24% and 18% of total HCVDB use, respectively. Decreased use of the HCVDB in the sustainment period is due to the decreased number of patients to screen, link to care, and treat. By the end of 2018 about 100,000 Veterans with HCV in VA care had been treated with HCV antiviral and 30,000 remained to be treated. The special populations report was used most in 2019 after a high proportion of Veterans had been treated and greater focus on more difficult to engage Veterans commenced.

On the Linkage to Treatment report there were 81,479 treatment assessments for 58,070 unique Veterans by 320 providers across 110 facilities in all 18 VA regions. Higher complexity facilities with more patients and in urban settings completed more writebacks than lowest complexity sites (947 vs 422, p = .003). The average number of comments per facility was 741±1211 (range 1–7010) and per user was 255±601 (range 1–6937). Individual Veterans had an average 1.4 writebacks (range 1–29) and had 0 to 1664 days between their first and final writeback (median 0; average 225). Among those assessed to not receive treatment, 19% were unable to be contacted, 16% refused treatment, 14% had unstable/uncontrolled comorbidities, 10% had documented ongoing nonadherence, 4% limited life expectancy, and 36% had other reasons (e.g., patient incarcerated, patient prefers non-VA care).

### User experience survey results

#### Respondents

A total of 105 surveys responses were returned from staff by the end of the implementation period (December 2018). Respondents represented each VA region and 79 VA facilities (61%). Most were clinical pharmacy specialists (32%), RN (19%), NP (19%), MD (15%), PA (6%) and other/unknown (9%). The average length of HCVDB use at the time of survey completion was 18±6 months. Most users were female (70%), with an average age of 45±11. Almost half (44%) had never previously used a PHM tool, 30% had used a local/regional dashboard, and 27% had used the Clinical Care Registry. Among survey respondents there were no differences in the number of list management actions or report executions by degree or specialty.

#### Popular dashboard features

Respondents reported using the following reports most frequently: Birth cohort screening (76%), SVR testing (75%), Linkage to care (69%), and On-Treatment (65%). Although differences in report use by degree did not reach statistical significance, advanced practice providers (NPs, PAs) and physicians were most likely to use the birth cohort screening report, compared to the linkage to treatment reports by clinical pharmacists, and the treatment/SVR report by RNs (Table 2). Non-clinical purposes of tracking progress and performance between clinics was least commonly used (31%).

**Table 2:** HCV report type use by user degree (n = 105).

	Birth Cohort (n = 80)	Linkage (n = 72)	Potential Treatment (n = 68)	On-Treatment (n = 79)
MD (n = 16)	81%	56%	63%	69%
PA/NP (n = 25)	88%	68%	64%	84%
PharmD (n = 35)	74%	80%	80%	69%
RN (n = 19)	68%	74%	74%	89%
Other (n = 10)	60%	40%	40%	60%

The following HCVDB features were rated in order of importance: find my patients easier (87%), obtain real-time updates (86%), score card to track how my facility is performing (80%), ability to add comments for patients (78%), sort my patients based on degree of liver disease 74%, ability to remove patients if no longer viremic (70%), see future appointments (64%), and score card to see how other facilities in my network are performing (59%).

#### Usability

The mean SUS score was 73±16 (range 38–100), corresponding to “good” usability higher than about 70% of all other technologies [16]. “Learnability” and “confidence” were high (81% and 76%, respectively) and only 10% reported needing the support of a technical person to use the HCVDB. Table 3 displays SUS item level mean scores and percent agreement.

**Table 3:** Usability and acceptability.

	mean (SD)	% Agreed
**System Usability Score (SUS)**	73 (16)	-
I like using the HCV Dashboard frequently.	4.10 (.91)	76%
I find the HCV Dashboard unnecessarily complex.	2.28 (1.0)	10%
I think the HCV Dashboard is easy to use.	4.00 (.88)	79%
I need the support of a technical person to be able to use this HCV Dashboard.	1.97 (.97)	10%
I found the various functions in the HCV Dashboard were well integrated.	3.86 (.83)	69%
I thought there was too much inconsistency in this HCV Dashboard.	2.42 (1.0)	14%
I would imagine that most people would learn to use this HCV Dashboard very quickly.	3.98 (.67)	81%
I find the HCV Dashboard very cumbersome to use.	2.09 (.90)	8%
I felt very confident using the HCV Dashboard.	3.98 (.96)	76%
I needed to learn a lot of things before I could get going with this HCV Dashboard.	2.27 (1.0)	12%
**Unified Theory of Acceptance and Use of Technology 2 (UTAUT2)**	-	-
*Performance Expectancy*	3.98 (.93)	-
I find the HCV Dashboard useful in my daily work.	4.02 (.97)	75%
Using the HCV Dashboard helps me accomplish things more quickly.	3.98 (.97)	75%
Using the HCV Dashboard increases my productivity.	3.95 (.99)	73%
*Social Influence*: People who influence my behavior think that I should use the HCV Dashboard.	3.84 (.81)	64%
*Facilitating Conditions*	3.80 (.80)	-
The HCV Dashboard is compatible with other technologies I use.	3.68 (.81)	57%
I can get help from others when I have difficulties using the HCV Dashboard.	3.91 (.92)	72%
*Price Value*: The HCV Dashboard is a good value for the effort.	4.21 (.90)	85%

SUS score was significantly associated with volume of HCVDB use (r = .40, p < .001). There was no relationship between the number of treatment assessment list management actions on the linkage report and perceived usability. There were also no differences in usability based on degree (p = .186), specialty (p = .295), HCVDB adoption timing (p = .091), or prior population health tool use (p = .254).

#### Acceptability

The overall UTAUT2 score was 3.98±.72 with difference by construct, in descending order: Price Value (4.21±.90), Performance Expectancy (3.98±.93), Social Influence (3.84±.81), and Facilitating Conditions (3.80±.80). Providers reported high Performance Expectancy, noting the HCVDB was useful in their daily work (75%), helped accomplish tasks more quickly (75%), and increased productivity (73%). Price Value of the HCVDB was high, with 85% agreeing it was a good value for the effort. Of Facilitating Conditions, most felt help was available if they had difficulties using the HCVDB (72%); fewer felt it was compatible with other technologies they used (57%). There were no differences in acceptability ratings by respondent characteristics.

Each UTAUT construct was positively associated with greater use (r = .48, p < .001) and SUS score (r = .67, p < .001). Acceptability was linked to two SUS usability constructs: 1) liking to use the HCVDB frequently, and 2) finding the various functions were well integrated. Conversely, SUS usability was linked to two acceptability constructs: 1) finding it useful in daily work, and 2) able to get help from others for difficulties using the HCVDB.

#### Free text responses

In total, 43 of 105 (41%) respondents provided a free text comment. About a third of comments were about possible design improvements, including faster page loading time, simpler export functions, and addressing consistency issues and ease of locating patients. Table 4 displays comments sorted by theme.

**Table 4:** Illustrative selection of free text comments.

Theme	Free Text Comment
**Improving care**	• “It’s the best thing that ever happened to HCV care in the VA.” (RN) • “It helped tremendously in our efforts to eradicate HCV. It is an incredibly valuable tool. Improved efficiency and outcomes.” (MD)
**Comparing to other tools**	• “HCV Dashboard compared to the old clinical case registry system is AMAZING! The dashboard is high tech, clean, organized and easy to use!” (NP) • “Much easier and better than our previous VISN [regional] dashboard” (PharmD)
**Population health**	• “It makes it very easy to get an overall idea of where you stand as a facility and where you need to go. It makes my life a lot easier.” (PharmD) • “I liked the ability to pull up any patient within our system whether viremic or not, screened or not. I really like to be able to see if they received DAA or labs from other sites as it improves my efficiency.” (RN)
**Distributing workload**	• “The dashboard is a great tool as many who were able to help in the efforts could sign on and start reviewing charts.” (RN) • “I like the fact that I can add comments as I do things in the patients review and the comments are always there to view and track historically what I have done for the patient” (NP)
**Staff Support**	• “The dashboard experts are outstanding in helping clinicians improve care, productivity and are always available to work on issues” (NP) • “I am very appreciative of the amazing work that has gone into the creation, development and maintenance of this dashboard and the feedback and quick responses from the dashboard team when questions have arisen.” (social work)
**Barriers**	• “At times the dashboard took a while to load which slowed my productivity.” (PharmD) • “I wish there was an easier way export dashboard data to programs like Excel to sort data when needed.” (PharmD) • “Most current version has integrated veterans that do not have active HCV. Makes it tedious to sift through veterans CPRS records to figure out if lost to follow up or care/or just at risk for disease.” (RN) • “We have used dashboard infrequently to try to increase outreach, but I don’t have personnel who are savvy enough or motivated enough to take on using it. We have our own treatment dashboard and database that helped us follow labs, track patients on treatment” (MD)
**Dashboard scale up**	• “I think it is absolutely essential to have this dashboard, especially with our efforts going to ALD and the different functions that the dashboard has.” (RN) • “I think the dashboard is absolutely essential to our success especially as we add ALD to our mission” (RN)

The most common theme of free-text comments was that the HCVDB improved HCV care efficiency and supported a volume of work that could have not otherwise been achieved. One pharmacist wrote, “*We could not have treated or screened the volume of patients that we have without the dashboard*! *It has been invaluable to our team*!” Other comments focused on the population health perspective: “*It makes it very easy to get an overall idea of where you stand as a facility and where you need to go*. *It makes my life a lot easier*.” For one respondent, one benefit was the ease of introducing new staff to the HCVDB: “*The dashboard is a great tool as many who were able to help in the efforts could sign on and start reviewing charts*.” However, another respondent remarked that they “don’t have personnel who are savvy enough or motivated enough to take on using it.” Several respondents noted that HCVDB staff were supportive and responsive to user questions: “*I am very appreciat[ive] of the amazing work that has gone into the creation*, *development and maintenance of this dashboard and the feedback and quick responses from the dashboard team when questions have arisen*.”

## Discussion

In 2016, responding to provider needs for a centralized and uniform PHM tool for HCV, a workgroup convened to create a national HCV dashboard in the VA. The cross-disciplinary user-centered design and development process allowed us to draw from prior versions of similar tools, identify user issues and make iterative improvements to create a functional product within four months. The HCVDB was a universally used tool with high usability and acceptability that allowed VA to achieve tremendous success in treating HCV.

Our study demonstrates how a user-centered design and ongoing user acceptance testing facilitated uptake and sustained use of the HCVDB across different user types. The HCVDB was created by users for users and later iterations were made with user feedback thus encouraging adoption and sustained use through a sense of ownership over its design. Understanding how patient data were consumed by providers and recognizing provider preferences were key factors in designing the HCVDB. This stakeholder engaged approach ensured the HCVDB matched the needs and capacities of users and enhanced the provider experience while reducing workload. This is consistent with literature showing the importance of visual simplicity in design and incorporating user feedback in developing electronic tools [17].

The HCVDB had rapid and widespread uptake across VA regions and individual facilities. Still, we found some variation in how the HCVDB was used, be it for increasing birth cohort testing, linking to care and treatment, monitoring treatment, or confirming cure. Generally, MDs and Advanced Practice Providers used the birth cohort screening reports most frequently, PharmDs the linkage and potential treatment reports, and RNs the SVR report. This use pattern reflects the rapidly changing roles in the first years of DAA implementation [18,19]. As others have reported, clinical pharmacists and advanced practice providers were central to DAA treatment initiation in recent years [20,21]. Clearer understanding of how providers integrated the HCVDB in care processes and the resulting efficiencies needs further study.

Although nearly half of the survey respondents had no prior PHM tool experience, the perceived usability and acceptability of the HCVDB was high. The HCVDB had a perceived usability greater than 70% of products tested in a 500-study review, suggesting it was situated between the level of usability of GPS and Microsoft Word and far greater than EMRs [22].

The combination of high “learnability” and low complexity contributed to high and continued use. High acceptability was driven by perceptions of perceived value, followed by beliefs around performance improvement, compatibility with other technologies, availability of supportive help and influential opinion leaders. Although Social Influence was the lowest scoring construct, this may be due to users already feeling highly self-motivated to use the tool. The degree of use was moderately associated with perceptions of usability and acceptability suggesting that even lower frequency users could have had strong user experiences. Most respondents found the HCVDB was instrumental in managing a high patient load and the influx in treatment, with some crediting the HCVDB as the key to sustaining high treatment volume. Early and ongoing stakeholder input and co-design may have supported such varied use and high user experience.

Several widescale infrastructure changes supported HCVDB implementation. First, VA is known for its centralized data repository, the Corporate Data Warehouse, from which data are pulled into the HCVDB and without which such a tool could not have been developed. A favorable policy environment and availability of DAAs to all Veterans supported rapid HCVDB development and implementation. The Collaborative infrastructure provided a forum in which to widely disseminate the HCVDB, provide training, and address user concerns. National metrics, guidelines, and policies strongly supported expanding treatment to all Veterans. Leadership support from VA operations, treatment advocacy, and the Collaborative unified providers across the country. Other data tools such as EMR clinical reminders for birth-cohort screening were also employed. Finally, treatment capacity was expanded by VA’s policies and hiring practices that allow non-physician providers to provide medical care to Veterans.

Key transferrable lessons included keeping information organized in compartments that are easily visualized and understood, limiting data to clinically significant information that informs reviews in the EMR, and list management to sort patient population data effectively. Provider-facing reports for population management for large cohorts of patients benefit from interactive functionalities such as capturing review comments. Incorporating business logic for color coding and flagging abnormal findings and adding functionalities to separate reviewed patients from those still needing to be reviewed reduced visual fatigue. Summary reports with performance metrics enhance self-motivated teams to rethink clinical processes and improve care along a continuum, including utilization of non-physician providers to breakdown tasks.

Tools such as the HCVDB can pivot healthcare systems to become proactive healthcare delivery models that maximize efficiency and resource utilization. Healthcare providers may feel optimistic about setting and achieving pragmatic targets while healthcare teams may recognize the important set of skills provided by different members of the team.

The HCVDB served as a proof of concept for ALD and hepatitis B dashboards, which are VHA goals [23]. Recent work has shown that high ALD dashboard utilizing facilities had higher hepatocellular carcinoma surveillance rates compared to lower- or non-user facilities [24].

### Limitations

This work has several limitations. The HCVDB was constructed within a large integrated healthcare system and at a time of restructuring HCV care processes; therefore, the implementation and evaluation described may not apply to other settings or to other health conditions. While this Dashboard evaluation was comprehensive and pulled from validated instruments, any survey study has the potential for sampling bias, whereby the people who choose to answer the survey are those with either the most or least favorable opinions. However, the respondents varied in their experience with the HCVDB and lengths of use, suggesting that we captured more than just “super-users.” Likewise, respondents did include suggestions for improvement in the free text. Another limitation is that, while high for provider surveys, there were a limited number of responses which impeded our ability to perform more complex statistical models of our findings. It is noteworthy that several networks abandoned their own dashboards in favor of the national dashboards–whether this occurred due to simplicity, ease of use or other reasons is unknown and beyond the scope of this paper (e.g., loss of local support to maintain a tool).

## Conclusions

The HCVDB was a central to VA’s successful HCV elimination efforts first by empowering clinicians to identifying Veterans with chronic HCV, it allowed providers to optimize linkage to curative treatments. User feedback served to provide developers with additional needs to close gaps in care and enabled providers to monitor treatments to ensure patients came back to confirm achievement of cure (SVR). The tool had rapid uptake, met provider needs, and scored highly on measures of usability, usefulness, and value. The HCVDB was essential to managing the US’ largest volume of patients with HCV, coordinating their care, and helping VA reach the goal of identifying and treating all Veterans with HCV. With widespread use of electronic records, dashboards and similar PHM tools stand to produce large-scale impact on care timeliness and efficiency.

Together, this information may be used to inform future dashboard development and to improve outcomes in disease-specific healthcare delivery.
